# The effect of nitrogen species on the catalytic properties of N-doped graphene

**DOI:** 10.1038/s41598-021-03403-8

**Published:** 2021-12-14

**Authors:** Malgorzata Skorupska, Anna Ilnicka, Jerzy P. Lukaszewicz

**Affiliations:** 1 Faculty of Chemistry, Nicolaus Copernicus University in Torun, Gagarina 7, 87-100 Toruń, Poland; 2 Centre for Modern Interdisciplinary Technologies, Nicolaus Copernicus University in Torun, Wilenska 4, 87-100 Toruń, Poland

**Keywords:** Chemistry, Materials science

## Abstract

The production of effective catalysts in the oxygen reduction reaction (ORR) continues to be a great challenge for scientists. A constant increase in demand for energy storage materials is followed by a proportionate increase in the number of reports on electrocatalyst synthesis. The scientific world focuses on environmentally friendly materials synthesized in accordance with the safest possible. In this work, we developed a facile method of obtaining heavy-metal-free electrode materials that are effective in ORR. Graphene-based catalysts were doped using azodicarbonamide (ADC) as the source of nitrogen, then carbonized at high temperatures in the range of 700–900 °C under inert gas flow. The produced materials were tested as catalysts for ORR, which is the most important reaction for Zn–air batteries and fuel cells. All obtained nitrogen-doped graphene foams showed increased catalytic activity in ORR owing to active sites created by nitrogen functional groups on the graphene surface. This paper shows that carbonization temperature has a significant impact on nitrogen content and that a small percentage of nitrogen may have a positive effect on the catalytic activity of the obtained materials. The number of transferred electrons in ORR was found to range from three to the maximal theoretical value, i.e., four.

## Introduction

Materials containing platinum are the best commercial electrocatalysts for the oxygen reduction reaction and serve as a paternal electrode material^1–3^; however, noble-metal-free electrode materials are an attractive alternative from an economical point of view and crucial for protecting the environment. Effective electrocatalysts in fuel cells^4,5^, lithium–air batteries^6,7^, and zinc–air batteries^8,9^ should be active in the oxygen reduction reaction (ORR)^10–12^, the key reaction in devices of this sort. Some very promising alternatives to noble-metal electrode materials are carbon materials doped with non-metal heteroatoms, e.g., phosphorus^13–15^, boron^14,16^ or nitrogen^14,17,18^ inserted into the basic carbon structure, of which the most promising catalysts are those containing nitrogen functional groups^19–21^, such as pyridine nitrogen (N-6) and pyrrole nitrogen (N-5). At the same time, very high concentrations of heteroatoms and a large amount of defects in the structure can contribute to a decrease in electronic conductivity and disadvantage the oxygen reduction reaction^22^. There are many nitrogen precursors that may be useful for synthesising N-doped carbon catalysts of high activity towards ORR, e.g., melamine^23,24^, urea^23,25^, adenine^23,26^, arginine^23,27^, or natural materials, such as green algae or gelatine^28,29^. We have demonstrated in our previous studies that such N-doped materials can be derived from amino acids^30^, chitin, and chitosan^31,32^, and provide high activity towards ORR. It has been shown that a large specific surface area, as well as a mesoporous structure, had a positive effect on ORR catalytic activity, this being due to good accessibility of catalytically active sites to the electrolyte and to the good charge transport provided by the carbon matrix. It was found that the influence of nitrogen content is not the key factor^18,33^.

To our understanding, the key challenge of any work in the field of N-rich carbon electrode materials is finding a non-platinum material with the ability to perform a four-electron reduction of an oxygen molecule. This feature is essential for the entire concept of applying N-rich carbons in fuel cells and reversible batteries (ORR on cathodes) as a replacement of the traditional Pt-loaded electrode materials. Among dozens of cited works, and hundreds or thousands left uncited, the occurrence of a four-electron reaction mechanism can only be attributed to single reports. Thus, the real novelty of our research attempt consists of a successful demonstration of the manufacturing protocol being able to gradually lead to that unique target. Another innovation of this study is the application of non-oxidized graphene rather than the graphene oxide and/or reduced graphene oxide which are used in the dominant synthesis approach in existing studies. The application of pristine graphene instead of oxidized graphene derivatives saves time and manufacturing costs.

In this paper, we continue the search for more effective non-metal ORR catalysts. It is our assumption that more efficient N-doping is achievable by means of more adequate N-precursors. In the current research, the influence of azodicarbonamide (ADC) and carbonization temperature on nitrogen content and ORR catalytic activity of nitrogen-doped graphene foam was investigated. Additionally, it was hypothesized that the general high nitrogen content is a not the sole steering parameter catalytic activity improvement, but that the type and concentration of pyridine nitrogen and pyrrolic nitrogen also plays a crucial role. Therefore, the content of such N-based functional groups was needs to be determined in context of and effective ORR process.

## Results and discussion

### Materials characterization

**Figure 1:**
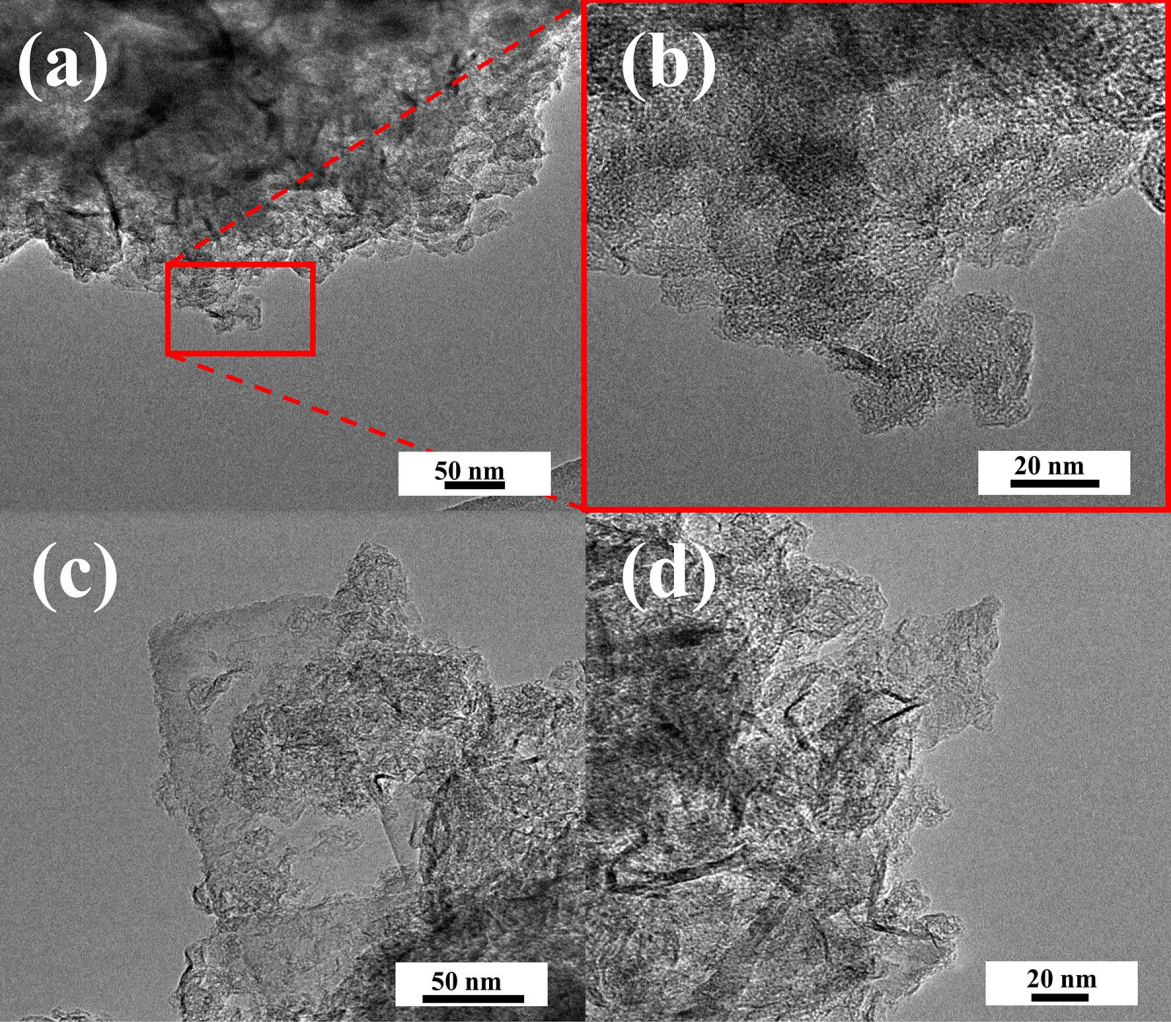
High-resolution transmission electron microscopy images of samples (**a**,**b**) 1-NGF-9, and (**c**,**d**) 2-NGF-9 at different magnifications.

While the structure of all obtained nitrogen-doped graphene foams was characterized using high resolution transmission electron microscopy (HRTEM) images, data for one sample is presented in this paper as fully representative of all obtained results. Figure 1 shows the structure of samples obtained with different weight ratios of reagents (1-NGF-9 and 2-NGF-9) and carbonized at 900 °C.

It is clearly visible that the structure is wrinkled, consisting of many layers superimposed on one another. Other materials show a similar wrinkled, flattened, and irregular structure. Calcium carbonate is assumed to form mesopores 20 nm in size, but the structure collapses when washed with hydrochloric acid, taking the form of graphene nanoplatelets (GNPs).

**Figure 2:**
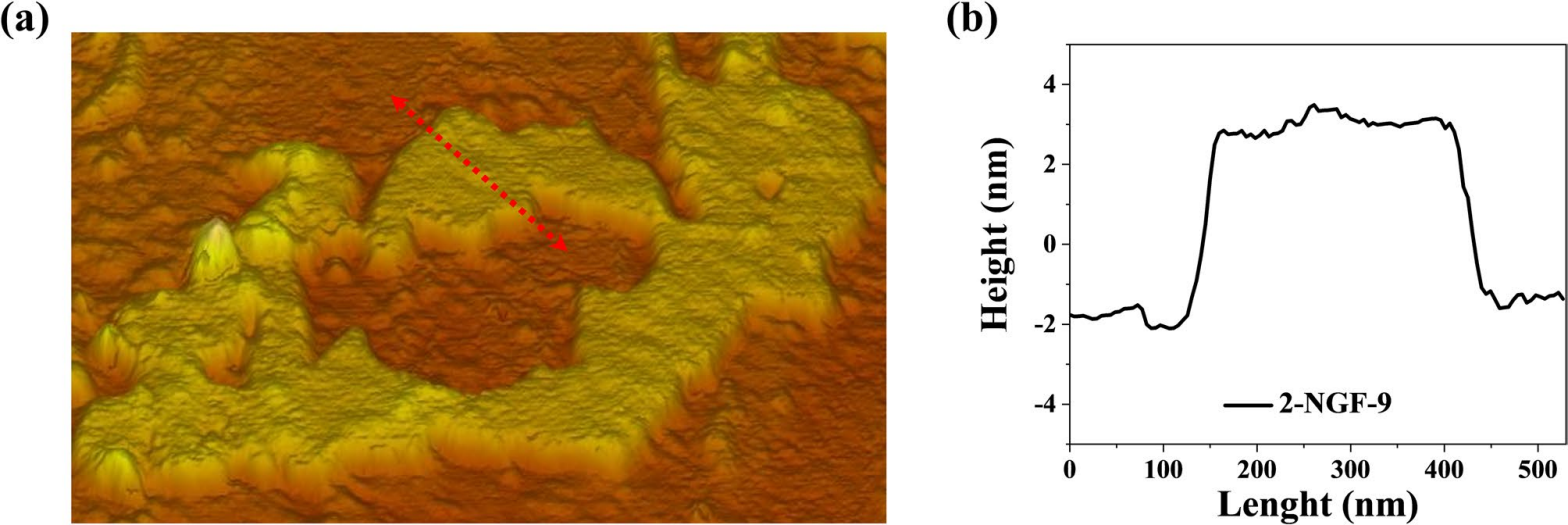
(**a**) AFM analysis highlighting the particular area, (**b**) height profile of marked area of 2-NGF-9.

Samples dispersed in ethanol were transferred to silicon wafers in order to study their surface structure and number of layers through the use of atomic force microscopy (AFM). Graphene sheets exhibit many contrasting regions that can be attributed to the layers of which they consist. When analyzing individual materials, all of them were found to exhibit many overlapping layers, which created a structured graphene material. The results are presented in Fig. 2. Results of Raman spectroscopy confirm that the obtained materials have many overlapping layers which are part of 3D structured graphene.

Elemental analysis was performed in order to investigate the obtained material’s nitrogen content and the effect of carbonization temperature on the content of carbon, hydrogen, and nitrogen. This study is important to confirming a previous conclusion stating that the content of nitrogen is not the only factor influencing ORR catalytic activity. The individual samples’ weight percentages of carbon, nitrogen, and hydrogen are presented in Table 1. The percentage of carbon was in a range of 82 to 93 wt% for all samples. For the 2-NGF-T series, increasing carbonization temperature is directly proportional to increasing carbon content, which indicates an improvement in the materials’ degree of graphitization. This trend is only in part maintained in the 1-NGF-T series, where the percentage was 86.25 wt% for a carbonization temperature of 700 °C, then decreased to 83.71 wt% for a carbonization temperature of 800 °C, and then increased to 92.94 wt% for 900 °C.

**Table 1:** Porosity parameters, nitrogen, hydrogen, and carbon content, and ratios of the intensities of G, D, and 2D-bands from the Raman spectra of 1-NGF-T and 2-NGF-T series.

Sample	Elemental content (wt%)			S_BET_ (m^2^ g^−1^)	V_t_ (cm^3^ g^−1^)	V_mi_ (cm^3^ g^−1^)	V_me_ (cm^3^ g^−1^)	I_D_/I_G_	I_2D_/I_G_
	C	H	N						
GNPs	87.32	0.90	0.72	750	0.99	0.13	0.86	0.64	0.40
1-NGF-7	86.25	1.77	1.83	635	0.82	0.12	0.70	0.95	0.34
1-NGF-8	83.71	0.94	2.35	620	0.76	0.14	0.62	0.83	0.43
1-NGF-9	92.94	0.72	1.02	657	0.84	0.12	0.72	0.81	0.37
2-NGF-7	82.31	1.86	3.01	640	0.80	0.13	0.67	0.68	0.39
2-NGF-8	87.60	0.95	3.18	533	0.74	0.11	0.63	1.05	0.34
2-NGF-9	93.09	0.67	0.95	562	0.69	0.13	0.56	0.40	0.46

The percentage of nitrogen is influenced not only by the type of precursor used, but by the carbonization temperature as well. For the series 1-NGF-T and 2-NGF-T, the highest nitrogen content was recorded for a carbonization temperature of 800 °C, in the amount of 2.35 wt% and 3.18 wt%, respectively. For the other samples from the 1-NGF-T series, carbonized at 700 °C and 900 °C, the percentage of nitrogen content was 1.83 wt% and 1.02 wt%, respectively. For samples from the 2-NGF-T series, carbonized at the two extreme temperatures of 700 °C and 900 °C, this content was at the level of 3.01 wt% and 0.95 wt%, respectively.

**Figure 3:**
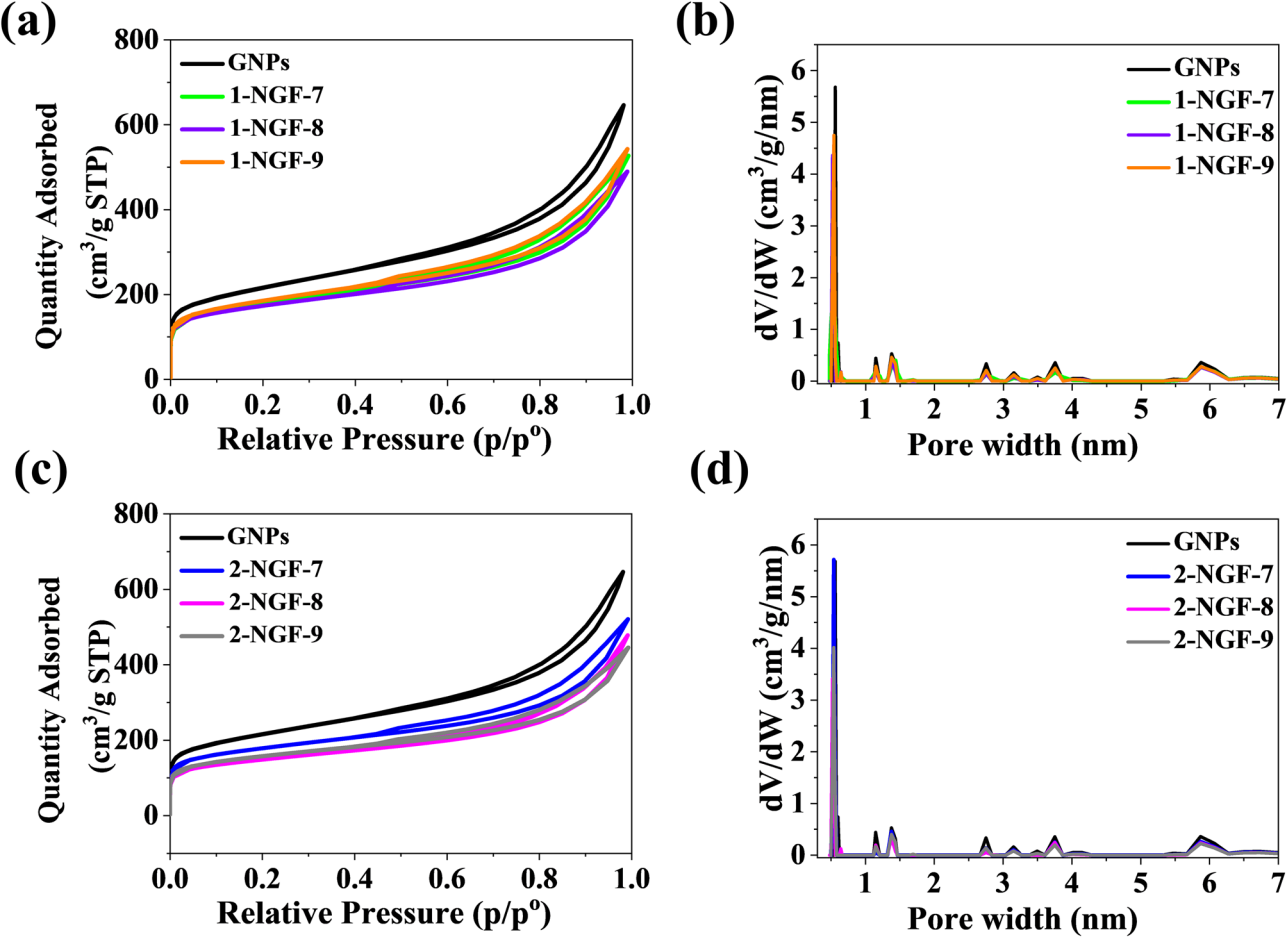
Nitrogen adsorption–desorption isotherms and NLDFT pore size distribution of samples in series (**a**,**b**) 1-NGF-T and (**c**,**d**) 2-NGF-T compared with GNPs.

In order to test the porosity of the samples and determine pore volume, a nitrogen adsorption–desorption analysis was performed. Figure 3a,c summarize the isotherms for all materials obtained and compare them to the GNPs to identify significant differences. According to the IUPAC classification^34^, all samples show the character of a type II isotherm, with a slightly pronounced hysteresis loop. The obtained materials did not show significant differences in relation to the GNPs, which suggests that the graphene structure remained stable after the synthesis process. A Brunauer–Emmett–Teller (BET) analysis of the 1-NGF-T series shows that the specific surface areas for the 1-NGF-7, 1-NGF-8, 1-NGF-9 samples was 635 m^2^ g^−1^, 620 m^2^ g^−1^, and 657 m^2^ g^−1^, respectively. The tested materials, 2-NGF-7, 2-NGF 8, 2-NGF-9, showed specific surface areas of 640 m^2^ g^−1^, 533 m^2^ g^−1^, and 562 m^2^ g^−1^, respectively. Thus, a slightly worse decrease in structural parameters (specific surface area and pore volume) was present for N-doped samples compared to the main carbon precursor (non-modified), i.e., graphene nanoplatelets (GNP).

In the second series, where the ADC was used in excess, a decrease in the specific surface area was observed with the increase in carbonization temperature, which could cause the graphene structure to collapse due to gases released in the carbonization process. Pore size distribution was determined based on the two-dimensional-non-localized density function theory (2D-NLDFT) method and is shown in Fig. 3b for the 1-NGF-T series and Fig. 3d for the 2-NGF-T series. For all the materials obtained, there were no significant changes in the pore size relative to the GNPs, showing micro and small mesopores. The lack of significant differences in the porous structure proves the strength of the material. The total pore volume (V_t_) for all materials obtained from both series did not exceed 1 cm^3^ g^−1^. Other parameters, such as micropore volume and mesopore volume, are presented in Table 1.

**Figure 4:**
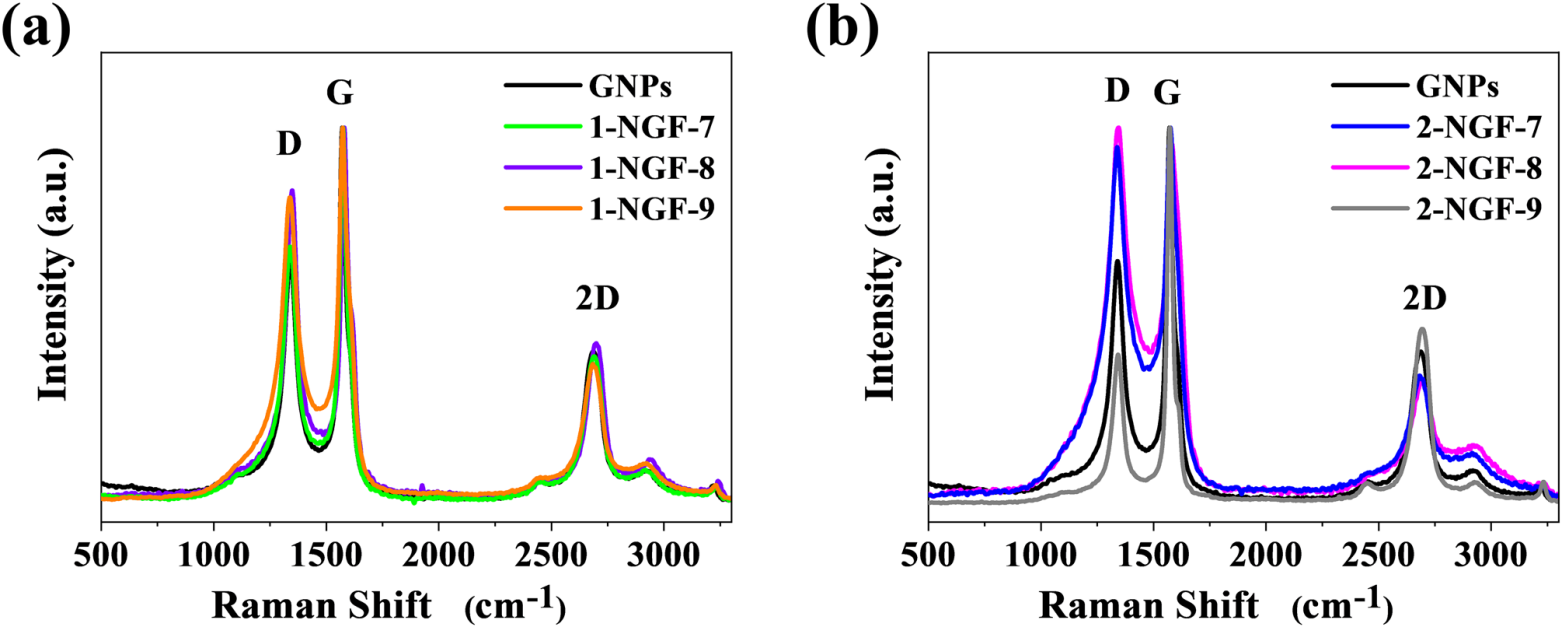
Raman spectra of samples in series (**a**) 1-NGF-T and (**b**) 2-NGF-T, compared with GNPs.

The quality of the graphene-based materials obtained and the degree of graphene layers/sheets association can be estimated using Raman spectroscopy (Fig. 4). All materials’ spectra contain three visible bands, D, G, and 2D, characteristic for graphene; the paternal Raman shifts for ideal graphene (for the 532 nm laser) are 1350 cm^−1^ for the D band, 1600 cm^−1^ for the G band, and 2700 cm^−1^ for the 2D band^35^. Raman spectra in Fig. 4a show the 1-NGF-T series compared to GNP’s spectrum. As is visible, the spectra overlap and there appear to be no particular changes with reference to the GNPs. The 2-NGF-T series is presented with reference material for GNPs in Fig. 4b. An increase in the D band intensity was observed for samples 2-NGF-7 and 2-NGF-8, which indicates that the introduced heteroatom content caused a defective structure. However, the intensity of the 2D band decreases with the increase of D band intensity. In the case of the 2-NGF-9 sample, the situation is completely different. The intensity of the 2D band increases concurrently with the D band. This indicates a qualitatively better material, with fewer defects and overlapping layers. The higher ratio of the intensity of the 2D and G bands in this sample, 0.46, proves that the material consists of many overlapping layers. Detailed parameters for the intensity and intensity ratios of D to G and 2D to G for all materials, as well as the GNPs, have been normalized and are presented in Table 1. All materials show a high degree of graphitization.

**Figure 5:**
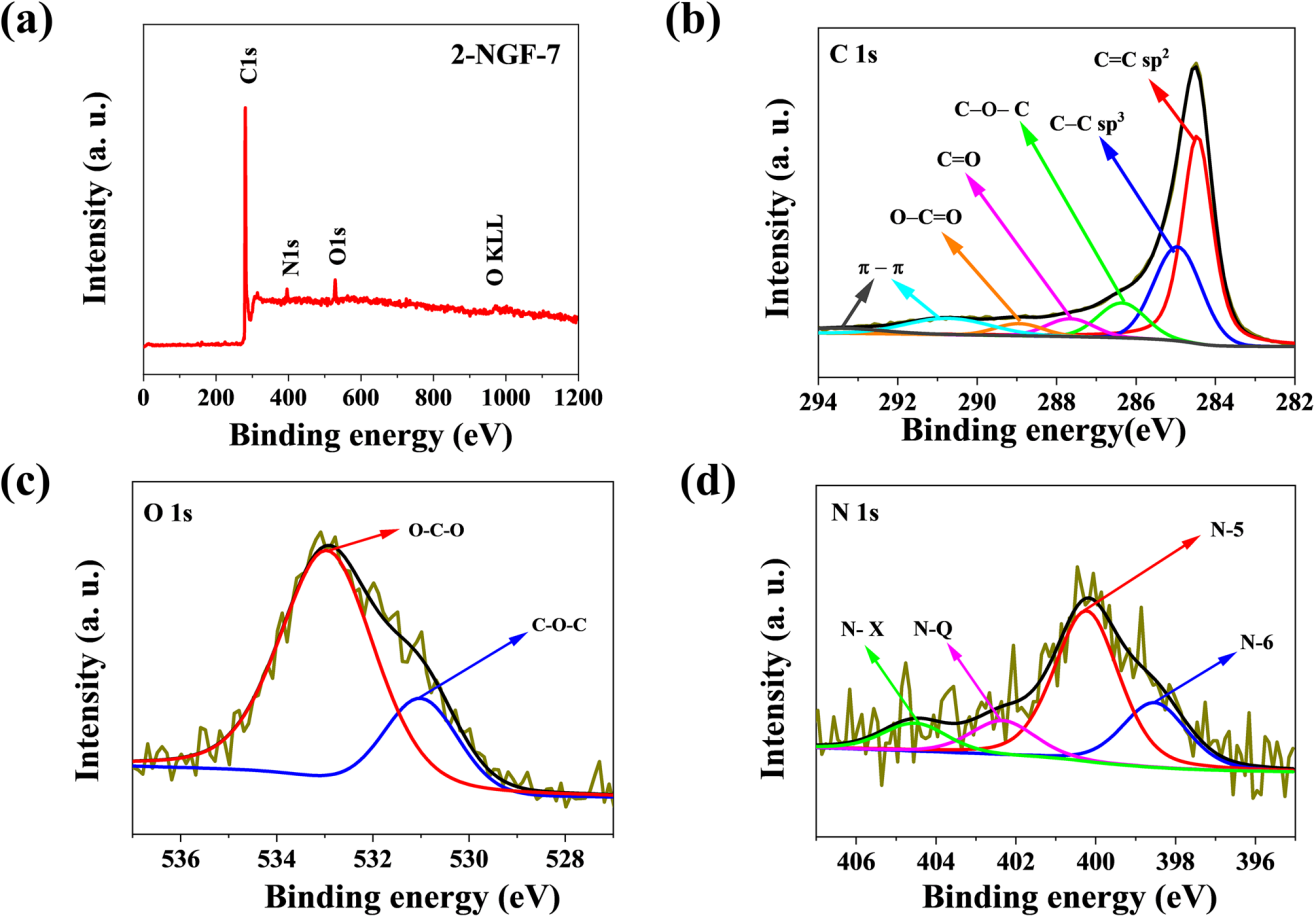
(**a**) The XPS survey spectrum and high resolution XPS spectra of (**b**) C1s, (**c**) O1s, and (**d**) N1s for the 2-NGF-7 sample.

As mentioned previously, the total nitrogen content is not the only factor influencing catalytic activity. The number of nitrogen functional groups on the edges of defective graphene structures is important. XPS measurements were made for a more in-depth analysis, which made it possible to determine the exact content of functional groups on the materials’ surfaces (Fig. 5a). We used azodicarbonamide as the nitrogen precursor, responsible for generating appropriate functional groups for ORR. Figure 5b–d show high-resolution XPS spectra for the 2-NGF-7 sample, which were characterized by three main elements: carbon, nitrogen, and oxygen.

All of obtained samples exhibit a similar structural content of carbon and oxygen atoms and the exact elemental composition of the respective nitrogen function groups is presented in Table 2. The C1s spectra for all samples consist of five types of bonds, located at 284.4 eV, 284.9 eV, 286.2 eV, 287.5 eV, 288.6 eV and corresponding to bonds of type C=C (sp^2^), C–C (sp^3^), C–O–C and/or C–NH, C=O (C-3), O–C–O, respectively^36^. Excitation of the shake-up type confirms the presence of C=C (sp^2^) bonds, related to aromatic forms, which appear at binding energies of 290.1 eV and 293.4 eV^36^. Based on the deconvolution of the N1s spectrum, it is possible to identify four peaks, characterized by bond energies at the values of 398.5 eV, 400.2 eV, and 402.3 eV, and 404.5 eV corresponding to the pyridine-nitrogen (N-6), pyrrolic-nitrogen (N-5), and graphitic-nitrogen (N-Q) groups, and N-oxides of pyridinic nitrogen (N-X), respectively^37^. The identified types of nitrogen functional groups should positively affect the catalytic properties of N-doped graphene foams for the oxygen reduction reaction. The content of N-5 and N-6 groups as a percentage of total N are shown in Table 2. Deconvolution of the O1s spectrum shows two types of oxygen bonds, at binding energies of 530.7 eV and 532.8 eV, corresponding to O*=C–O and/or O–C–O and O=C–O* and/or C–O–C, respectively^36,38^.

**Table 2:** Elemental composition of N-doped graphene foams determined by XPS analysis.

Sample	Total C (at.%)	Total O (at.%)	Total N (at.%)	Nitrogen functional group (at. %)				(N-5) and (N-6) (% relative to total N)
				N-6	N-5	N-Q	N-X	
1-NGF-7	94.1	3.9	2.1	0.2	1.3	0.3	0.3	71
1-NGF-8	93.5	4.1	2.2	0.4	1.5	0.2	0.1	86
1-NGF-9	95.0	3.7	1.0	0.1	0.8	0.0	0.1	90
2-NGF-7	93.4	3.9	2.7	0.7	1.5	0.3	0.2	81
2-NGF-8	94.6	3.1	1.9	0.5	1.2	0.1	0.1	89
2-NGF-9	94.7	4.2	0.7	0.2	0.5	0.0	0.0	100

### Electrochemical performance

**Figure 6:**
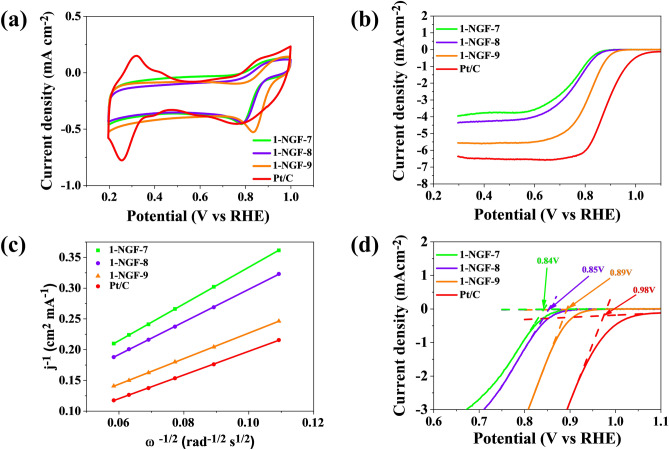
The results of electrochemical performance for 1-NGF-T and Pt/C in saturated-O_2_ 0.1 mol L^−1^ KOH (**a**) CV plots with a scan rate of 10 mV s^−1^_,_ (**b**) LSV plots with a scan rate of 5 mV s^−1^ and rotation speed of 1600 rpm, (**c**) Koutecky–Levich plots at 0.5 V vs RHE, (**d**) onset potential for samples in the 1-NGF-T series.

**Figure 7:**
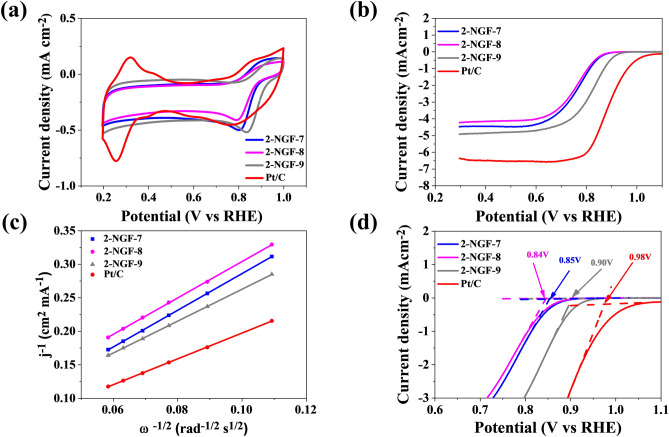
The results of electrochemical performance for 2-NGF-T compared with Pt/C in saturated-O_2_ 0.1 mol L^−1^ KOH (**a**) CV plots with a scan rate of 10 mV s^−1^_,_ (**b**) LSV plots with a scan rate 5 mV s^−1^ and rotation speed of 1600 rpm, (**c**) K–L plots at 0.5 V vs RHE, (**d**) onset potential for samples in 2-NGF-T series.

Electrochemical tests were used order to determine the application potential of the obtained nitrogen-doped graphene foams. Materials were tested as electrocatalysts for ORR to assess whether any given one is suitable for use in electrical devices, such as zinc–air batteries or fuel cells. Electrochemical methods, cyclic voltammetry (CV) and linear voltammetry (LSV), were used, then the obtained results were compared with the commercial Pt/C material. The four-electron oxygen reduction reaction is the most desirable path and allows the effective operation of devices based on ORR. Tests were carried out in an oxygen-saturated alkaline electrolyte. The obtained CV and LSV results are shown in Fig. 6 (series 1-NGF-T) and Fig. 7 (2-NGF-T series). The number of electrons transferred in ORR was calculated from the K–L equations and presented as a function of current density (j^−1^) at a spin speed ω of 0.5 V vs RHE (Fig. 6c). All materials showed increased catalytic activity, as can be observed in the CV voltammograms; detailed data are presented in Table 3. Analysis of the first series, 1-NGF-T, indicates that the sample carbonized at 900 °C (1-NGF-9) was the most effective in ORR. The cathode peak (Fig. 6a) shifted from the more positive values was at 0.83 V vs reversible hydrogen electrode (RHE). Linear voltammetry at 1600 rpm and a scan rate of 0.005 mV s^−1^ (Fig. 6b,d) shows an onset potential of 0.89 V vs RHE. The shape of the LSV curve for 1-NGF-9 is very close to that of the commercial Pt/C catalyst and shows a high diffusion-limiting current of 5.30 mA cm^−2^. The remaining materials from the 1-NGF-T series had lower catalytic activity compared to sample 1-NGF-9, carbonized at 900 °C, which exhibited a four-electron oxygen reduction reaction. The highest catalytic activity in relation to the remaining materials stems from two factors. One of them is the extensive structure of mesopores (0.72 cm^3^ g^−1^, higher than in all others), which further enhances the availability of the electrolyte and thus facilitates the diffusion of electrons in ORR. The other condition that this sample accomplished is the percentage share of N-5 and N-6 groups in the total nitrogen content, as follows from the XPS analysis, which has a beneficial effect on the oxygen reduction reaction^39^.

**Table 3:** ORR performance parameters of obtained N-doped graphene foams compared to commercial Pt/C catalyst, tested in 0.1 mol L^−1^ KOH.

Catalyst	E_p_ (V vs RHE)	E_onset_ (V vs RHE)	E_1/2_ (V vs RHE)	Diffusion-limiting current (mA cm^−2^)	n (0.5 V)
Pt/C	0.76	0.98	0.88	6.37	4.00
1-NGF-7	0.78	0.84	0.75	3.80	3.04
1-NGF-8	0.79	0.85	0.76	4.16	3.43
1-NGF-9	0.83	0.89	0.82	5.30	4.00
2-NGF-7	0.80	0.85	0.76	4.50	3.32
2-NGF-8	0.79	0.84	0.76	4.06	3.35
2-NGF-9	0.83	0.90	0.82	4.70	3.82

**Figure 8:**
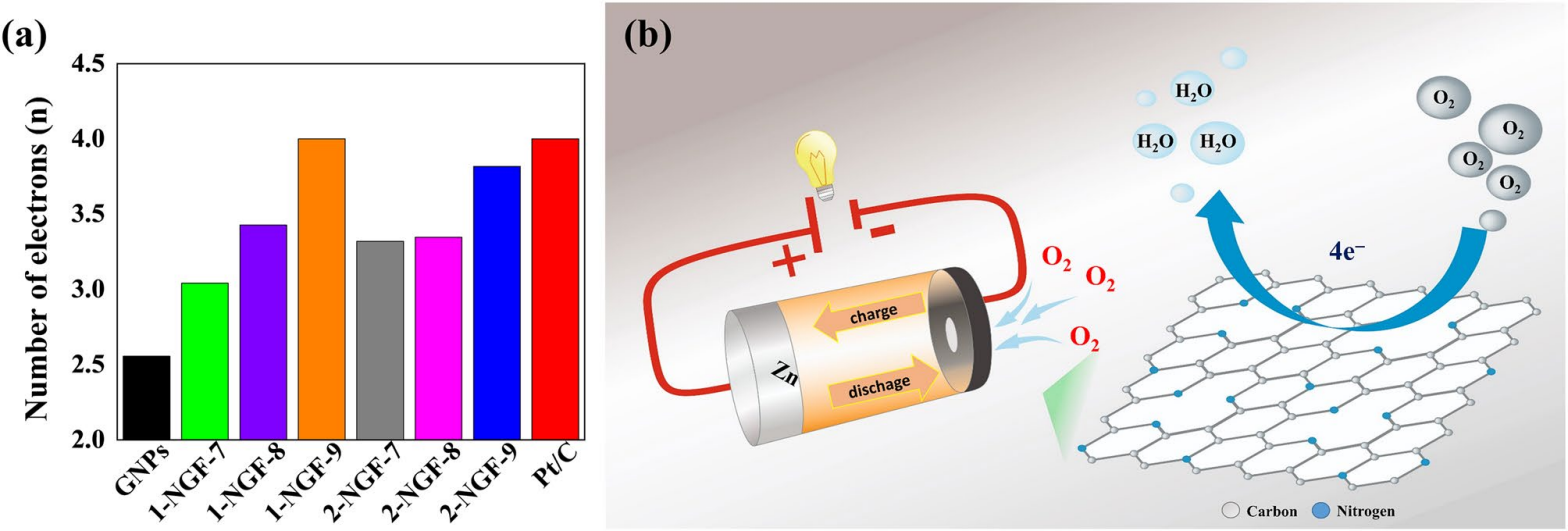
(**a**) The number of transferred electrons in the 1-NGF-T and 2-NGF-T series compared to the commercial catalyst Pt/C and GNPs. (**b**) The schematic diagram of the four-electron oxygen reduction reaction crucial for rechargeable zinc–air battery.

The number of transferred electrons for samples 1-NGF-7 and 1-NGF-8 was 3.04 and 3.43, respectively (Fig. 8). The pyridine and pyrrole functional groups, despite their low content located at the edges, react with hydroxyl groups derived from alkaline forms of the electrolyte, creating an active site at the same time. The 2-NGF-T series also showed increased catalytic activity in ORR, as indicated by the CV curves in Fig. 7a, where there are clearly visible cathode peaks for materials carbonized at 700 °C, 800 °C, and 900 °C, shifted relative to the positive values and amounting to 0.81, 0.79, and 0.83 V vs RHE, respectively. The material carbonized at 700 °C showed a very high current density in CV measurements compared to other samples in this series. Similar values of the initial potential and the diffusion-limiting current indicate little differentiation of the material, however, they show catalytic activity in relation to the GNPs, with their transferred-electron numbers were 3.32, 3.35, and 3.82 for the 2-NGF-7, 2-NGF-8, and 2-NGF-9 samples, respectively. The high increase in catalytic activity of the obtained materials as compared to the GNPs is due to active functional groups deposited on the edges of the graphene sheets. These kinds of nitrogen groups strongly influence the oxygen reduction reaction^40,41^ mechanism. The catalytic activity of 1-NGF-9 and 2-NGF-9 samples can be explained based on the paper by Guo et al., where the authors suggest that pyridinic nitrogen determines electrocatalytic activity^41^. The authors describe that the Lewis base carbon atoms neighboring pyridinic nitrogen, not pyridinic nitrogen itself, are electrocatalytic ORR centers in N-doped carbon materials. Furthermore, pyrrolic nitrogen species are responsible for ORR performance in an alkaline medium^42,43^. Therefore, the combined effect of these two groups (pyridinic nitrogen and pyrrolic nitrogen) in samples 1-NGF-9 and 2-NGF-9, with the highest concentration (N-6 and N-5) of the total nitrogen content (90% and 100%), is related to the highest number of transferred electrons.

Figure 8a summarizes the number of transferred electrons in the oxygen reduction reaction of the obtained materials, the GNPs, and Pt/C. All materials obtained with the proposed method showed catalytic activity. The four-electron oxygen reduction reaction (Fig. 8b) is facilitated by the quality of nitrogen groups, not the percentage of nitrogen, which affects the rapid reaction kinetics at the edges of multilayer graphene. Our results support the notion that the total nitrogen percentage is not directly proportional to catalytic activity^44^ and obtained materials are promising electrocatalysts for zinc–air battery.

**Figure 9:**
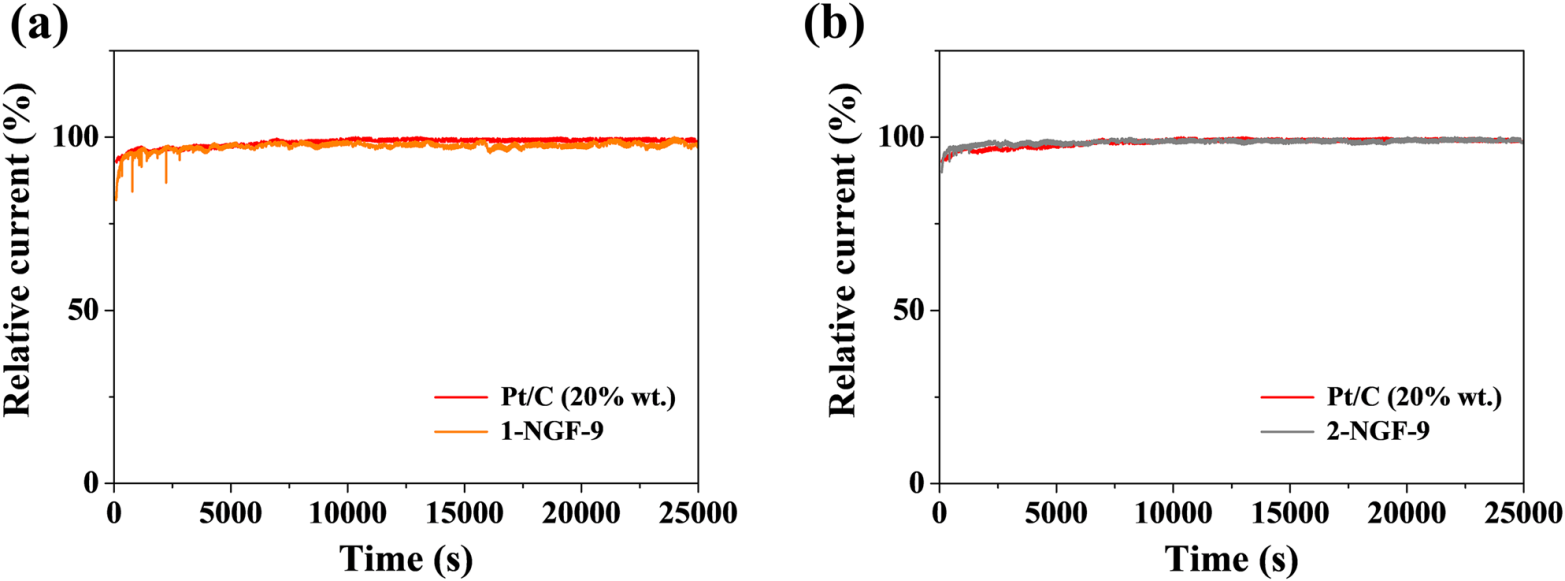
Chronoamperometric responses of (**a**) 1-NGF-9 and (**b**) 2-NGF-9 compared to a Pt/C electrode in O_2_-saturated 0.1 M KOH solution at 0.5 V vs RHE.

From an economic point of view, it is important for the production of energy storage devices and metal-air batteries to obtain materials with comparable stability to the commercial platinum-based materials. The stability of materials in the oxygen reduction reaction is one of the most important parameters when evaluating them for potential applications and making attempts to eliminate heavy metals from commercial materials. Chronopotentiometry tests were carried out to estimate the durability of the 1-NGF-9 (Fig. 9a), 2-NGF-9 (Fig. 9b), and Pt/C electrodes. After a long test of 25,000 s, all samples still remained at a high current stability compared to commercial Pt/C.

## Materials and methods

### Sample preparation

To prepare nitrogen-doped graphene foams, first carrageenan (1 g) was dissolved in deionized water (30 ml). Then, graphene nanoplatelets (1 g) were mixed with calcium carbonate (1 g) nanoparticles (average particle size 15–40 nm) and dispersed in the carrageenan solution. Lastly, azodicarbonamide (1 g) used as a nitrogen source. Pore-forming material was also added to the obtained solution. This was then mixed for 30 min on a magnetic stirrer, and after this time, the samples were lyophilized for 24 h. The obtained dry mass was carbonized at 700 °C, 800 °C, or 900 °C under the flow of inert gas N_2_ at a heating rate of 10 °C min^−1^ and kept at the maximal temperature for 1 h. Since the melting point of ADC is 225 °C, this component was completely removed from the sample during the high-temperature carbonization process. Samples were treated with concentrated HCl (36–38%) for 20 min to remove calcium carbonate and washed with distilled water to the point of neutral pH. The resultant products were denoted as 1-NGF-T and 2-NGF-T, where: 1 and 2 describe the mass ratio of reagents CaCO_3_:ADC of 2:1 and 1:2, respectively; NGF stands for nitrogen-doped graphene foam; T is the carbonization temperature of 700 °C, 800 °C, and 900 °C, indicated by 7, 8, and 9, respectively.

### Physicochemical characterization

A high-resolution transmission electron microscope (HRTEM FEI Tecnai F20 X-Twin, Brno, the Czech Republic) was used to observe the structure of the samples at an accelerating voltage of 200 kV. Raman spectra were obtained using 532 nm laser excitation (microscope InVia Renishaw, Renishaw Company, Gloucestershire, Great Britain). The specific surface area and pore size distribution were ascertained from adsorption and desorption isotherms measured at 77 K by means of an automatic volumetric analyzer (ASAP 2020 Plus, Micromeritics, Norcross, USA). The Brunauer–Emmett–Teller (BET) method was used to determine the specific surface area (S_BET_). The pore size distribution was calculated using the two-dimensional-non-localized density functional theory method. The single-point total pore volume (V_t_) for the obtained materials was measured at the maximum relative pressure of p/p_o_. The micropore volume (V_mi_) was determined using the t-Plot method, while the mespopore volume (V_me_) was calculated by subtracting V_mi_ from V_t_. Information about the elemental composition (carbon, nitrogen and hydrogen) of the nitrogen-doped graphene foams was collected using elemental combustion analysis (Vario MACRO CHN, Elementar Analysensysteme GmbH, Germany). The atoms’ chemical state was identified through X-ray photoelectron spectroscopy (XPS) measurements with a VG Scientific ESCALAB-210 (Japan), photoelectron spectrometer with Al Ka radiation (1486.6 eV). Using the Scanning Probe Microscope (SPM) by Veeco (Digital Instrument, USA), the obtained materials were tested in order to determine the values of graphene thickness using atomic force microscopy.

### Electrochemical measurements

In order to determine the electrochemical properties of the obtained nitrogen-doped graphene foams, their electrochemical activity was assessed using a rotating disc electrode (RDE) on an Autolab electrochemical analyzer (PGSTAT128N, the Netherlands). In the conventional three-electrode system, the obtained carbon material on a glassy carbon electrode (GCE with a diameter of 3 mm) was used as a working electrode, Ag/AgCl in 3 mol L^−1^ KCl was used as the reference electrode, a platinum wire was used as the counter electrode, and an aqueous solution of 0.1 mol L^−1^ KOH was used as the electrolyte. Platinum-based commercial carbon, Pt/C (20 wt% of Pt), was used as a reference catalyst. The preparation of ink is based on mixing 2.5 mg of the catalyst and its dispersion in 0.55 ml of a mixture of distilled water, ethanol, and Nafion (0.5 wt% of Nafion) for 60 min. The prepared ink was applied to a glassy carbon electrode previously polished by a diamond and alumina polishing pad. Cyclic voltammetry (CV) and linear sweep voltammetry (LSV) measurements were carried out to determine oxygen reduction reaction activity in the obtained nitrogen-doped foams. The CV curves were measured at a scan speed of 10 mV s^−1^, while the LSV curves were measured at 5 mV s^−1^ and with a rotation speed of 1600 rpm. The 0.1 mol L^−1^ KOH electrolyte solution was saturated with oxygen and nitrogen before CV and LSV measurement. The number of electrons (n) was calculated from the Koutecky–Levich (K–L) equation, based on a full LSV measurement at 0.5 V vs reversible hydrogen electrode (RHE):

1 $$ {\text{J}}^{ - 1} = {\text{ J}}_{{\text{L}}}{^{ - 1}} + {\text{ J}}_{{\text{K}}}{^{ - 1}} =  \left( {{\text{B}}\upomega^{1/2} } \right)^{ - 1} + {\text{ J}}_{{\text{K}}}{^{ - 1}} $$

2 $$ {\text{B}} = 0.62{\text{nFC}}_{0} \left( {{\text{D}}_{0} } \right)^{2/3} \upnu^{ - 1/6} $$

where: J is assigned to the measured current density, J_L_ is defined as the current density limiting diffusion, and J_K_ is the kinetic current density; ω is the angular velocity of the electrode; n is the number of direct electrons involved in the reaction; F indicates Faraday's constant (96,485 C mol^−1^); C_0_ denotes the dissolved oxygen concentration and is equal 1.2 * 10^−6^ mol L^−1^ in 0.1 mol L^−1^ KOH; D_0_ is equal to the diffusion coefficient of dissolved oxygen, 1.9 * 10^−5^ cm^2^ s^−1^ in 0.1 mol L^−1^ KOH; ν for 0.1 mol L^−1^ KOH is 0.01 cm^2^ s^−1^ and is defined as the kinetic viscosity of the electrolyte. A determination of the number n is possible when both equations are used to calculate the slope of the K–L curve.

## Conclusions

In conclusion, the obtained nitrogen-doped graphene foams are environmentally friendly, metal-free electrocatalysts with properties comparable to a Pt-based catalyst. The azodicarbonamide used here is an effective precursor of nitrogen atoms, especially N-5 and N-6 groups. The total content of nitrogen functions N-5 and N-6, which in all samples were significant for ORR, was above 82%. The best electrochemical performance was exhibited by the 1-NGF-9 and 2-NGF-9 samples, carbonized at 900 °C, showing a four-electron oxygen reduction in an alkaline medium despite the lowest nitrogen content. The materials produced here can be successfully used as electrode components in metal–air batteries or fuel cells as some of them are capable of a four-electron reduction of oxygen molecules, with this feature remaining constant in conditions simulating repeatable usage of a zinc–air battery. Further research on surface area development or the introduction of dual heteroatom doping into the graphene structure may expand these materials’ range of applications beyond electrode materials for batteries and into supercapacitors or photovoltaic systems.
